# Performance of the COVID19SEROSpeed IgM/IgG Rapid Test, an Immunochromatographic Assay for the Diagnosis of SARS-CoV-2 Infection: a Multicenter European Study

**DOI:** 10.1128/JCM.02240-20

**Published:** 2021-01-21

**Authors:** Mario Plebani, Marijo Parčina, Issam Bechri, Gianguglielmo Zehender, Vedrana Terkeš, Balqis Abdel Hafith, Spinello Antinori, Sylvie Pillet, Sylvie Gonzalo, Achim Hoerauf, Alessia Lai, Miro Morović, Thomas Bourlet, Alessandro Torre, Bruno Pozzetto, Massimo Galli

**Affiliations:** a Department of Medicine-DIMED, University of Padova, Padua, Italy; b Department of Laboratory Medicine, University Hospital of Padova, Padua, Italy; c Institute of Medical Microbiology, Immunology and Parasitology, University Hospital Bonn, Bonn, Germany; d Department of Infectious Agents and Hygiene, University Hospital of Saint-Etienne, Saint-Etienne, France; e Department of Biomedical and Clinical Sciences Luigi Sacco, University of Milan, Milan, Italy; f Department of Infectious Diseases, Zadar Regional Hospital, Zadar, Croatia; g GIMAP EA3064, Faculty of Medicine, University Jean Monnet of Saint-Etienne, Saint-Etienne, France; Cepheid

**Keywords:** COVID-19, SARS-CoV-2, immunochromatographic assay, multicenter, rapid test, serology

## Abstract

This study assessed the diagnostic performance of the new COVID19SEROSpeed IgM/IgG rapid test (BioSpeedia, a spinoff of the Pasteur Institute of Paris) for the detection of antibodies against severe acute respiratory syndrome coronavirus 2 (SARS-CoV-2) in comparison to other commercial antibody assays through a large cross-European investigation. The clinical specificity was assessed on 215 prepandemic sera (including some from patients with viral infections or autoimmune disorders). The clinical sensitivity was evaluated on 710 sera from 564 patients whose SARS-CoV-2 infection was confirmed by quantitative reverse transcription-PCR (qRT-PCR) and whose antibody response was compared to that measured by five other commercial tests. The kinetics of the antibody response were also analyzed in seven symptomatic patients. The specificity of the test (BioS) on prepandemic specimens was 98.1% (95% confidence interval [CI], 96.2% to 99.4%). When tested on the 710 pandemic specimens, BioS showed an overall clinical sensitivity of 86.0% (95% CI, 0.83 to 0.89), with good concordance with the Euroimmun assay (overall concordance of 0.91; Cohen’s kappa coefficient of 0.62). Due in part to simultaneous detection of IgM and IgG for both S1 and N proteins, BioS exhibited the highest positive percent agreement at ≥11 days post-symptom onset (PSO). In conclusion, the BioS IgM/IgG rapid test was highly specific and demonstrated a higher positive percentage of agreement than all the enzyme-linked immunosorbent assay/chemiluminescence immunoassay (ELISA/CLIA) commercial tests considered in this study. Moreover, by detecting the presence of antibodies prior to 11 days PSO in 78.2% of the patients, the BioS test increased the efficiency of the diagnosis of SARS-CoV-2 infection in the early stages of the disease.

## Abstract (precis)

This study assessed the diagnostic performance of the new coronavirus disease 2019 (COVID-19) SEROSpeed IgM/IgG Rapid Test (BioSpeedia, a spinoff of the Pasteur Institute of Paris) for the detection of antibodies against severe acute respiratory syndrome coronavirus 2 (SARS-CoV-2) in comparison to other commercial antibody assays through a large cross-European investigation. The clinical specificity was assessed on 215 prepandemic sera (including some from patients with viral infections or autoimmune disorders).

## INTRODUCTION

Severe acute respiratory syndrome coronavirus 2 (SARS-CoV-2) infection represents a relevant diagnostic challenge to the health care community (1). It has been widely reported that this infection may result in mild to totally asymptomatic disease in the majority of individuals, although it can progress into a severe pneumonia that often needs artificial oxygenation and/or mechanical ventilation, with a proportion of patients who die (2). The incubation period of coronavirus disease 2019 (COVID-19) ranges between 2 and 14 days, with most cases manifesting within 3 to 6 days. In symptomatic cases, the onset of symptoms occurs within 14 days after the infection and on average in 5 days (3).

Assays based on quantitative reverse transcription-PCR (qRT-PCR) detecting SARS-CoV-2 RNA yield positive results in upper respiratory samples from symptomatic cases within a few days prior to the onset of symptoms and can remain positive for several weeks after the end of symptoms (4, 5). However, although considered the reference method for the diagnosis of SARS-CoV-2 infection, these tests exhibit a significant number of false-negative results depending on infection stage, viral load or quality, and type of sampling (6–10). Although the diagnostic value of antibody determination is limited in the first 2 weeks of the disease, a time period which in many patients coincides with the window phase prior to seroconversion (8), the use of serological tests has been suggested also in the first week after the onset of symptoms as complementary diagnostic tools to enhance the sensitivity and accuracy of laboratory diagnosis (5, 8, 11, 12). In one study, the combined use of an RT-PCR test and an IgM ELISA increased the accuracy of the diagnosis for SARS-CoV-2 from 51.9% to 98.6% (13). Therefore, serological tests are useful to identify previous infections but also to confirm the presence of current infection or to catch infections missed by RT-PCR. With adequate sensitivity and specificity, serological tests may also be useful for serosurveillance programs, particularly in high-risk subpopulations (e.g., health care workers), for the containment of new outbreaks and in seroprevalence studies (14). Currently, levels of antibodies are predominantly measured by enzyme-linked immunosorbent assay (ELISA) and chemiluminescence assay (CLIA) technologies. These methods, although valid, require the time and organization necessary for venous sampling as well as specific and sometimes sophisticated and costly instruments and skilled personnel. In contrast, point-of-care testing (POCT) by lateral flow assays (LFAs) may facilitate SARS-CoV-2 diagnosis. In fact, these tests are designed to be rapid, sensitive, highly accessible, and easily performed, requiring only a small amount of blood. At the time of writing, several hundred candidate POCTs were under evaluation for their applicability in identifying SARS-CoV-2-infected individuals. Some of them have been shown to deliver good analytical performances (15, 16), whereas many others exhibited low positive or negative percent agreement (17).

The aim of this cross-European study involving 5 laboratories in Germany, France, and Italy was to evaluate the diagnostic performance of the COVID19SEROSpeed IgM/IgG rapid test (abbreviated “BioS” here) for the detection of antibodies against SARS-CoV-2 in comparison to those of well-established commercial ELISAs and CLIAs and to analyze the kinetics of antibody response with reference to onset of symptoms in severe and nonsevere COVID-19.

## MATERIALS AND METHODS

### COVID19SEROSpeed IgM/IgG test.

The BioS test evaluated in this study was developed by the BioSpeedia Company (Paris, France), a spinoff of the Pasteur Institute of Paris. It consists of an immunochromatographic LFA rapid test specifically designed to detect antibodies directed to the S1 spike protein as well as the nucleocapsid protein of SARS-CoV-2. The test can be performed either on a fingerstick sample or on serum or plasma specimens. After deposition of 40 μl of patient sample in the test cartridge, one drop of buffer furnished in the kit must be added immediately to start migration of the sample. The reading must be performed visually within 15 min. In addition to a control line confirming that the presence of the serum specimen was actually detected, two bands allow the separate detection of specific IgM and IgG antibodies. The assay was used by the participating centers according to the manufacturer’s instructions. The result for each class of antibodies was determined as negative or positive based on visual inspection. In the absence of the control line, the test was interpreted as invalid.

### Participating centers.

The study was conducted from February to June 2020 in 5 hospitals located in 3 European countries severely impacted by the COVID-19 pandemic, including three in Northern Italy (Azienda Socio Sanitaria Territoriale [ASST] Fatebenefratelli-Sacco, University Hospital of Milan; University Hospital of Padova; ASST Spedali Civili di Brescia, University of Brescia), one in France (University Hospital of Saint-Etienne), and one in Croatia (University Hospital of Zadar), for which samples were processed at the University Hospital Bonn (Germany). The protocol of the study was approved by the Ethical Committee of each center.

### Serum specimens.

Prepandemic serum specimens (*n* = 215) were used to check the specificity of the test and included 21 samples from patients with recent infection by different infectious agents (Toxoplasma gondii, Mycoplasma pneumoniae, measles virus, cytomegalovirus, HIV, and hepatitis B virus) and 20 samples from patients exhibiting various autoantibodies, e.g., rheumatoid factors, antibodies directed to myeloperoxidase, proteinase 3, or native or extractable nuclear antigens. These samples had been collected in 1999 and kept frozen at −30°C until use.

**FIG 1:**
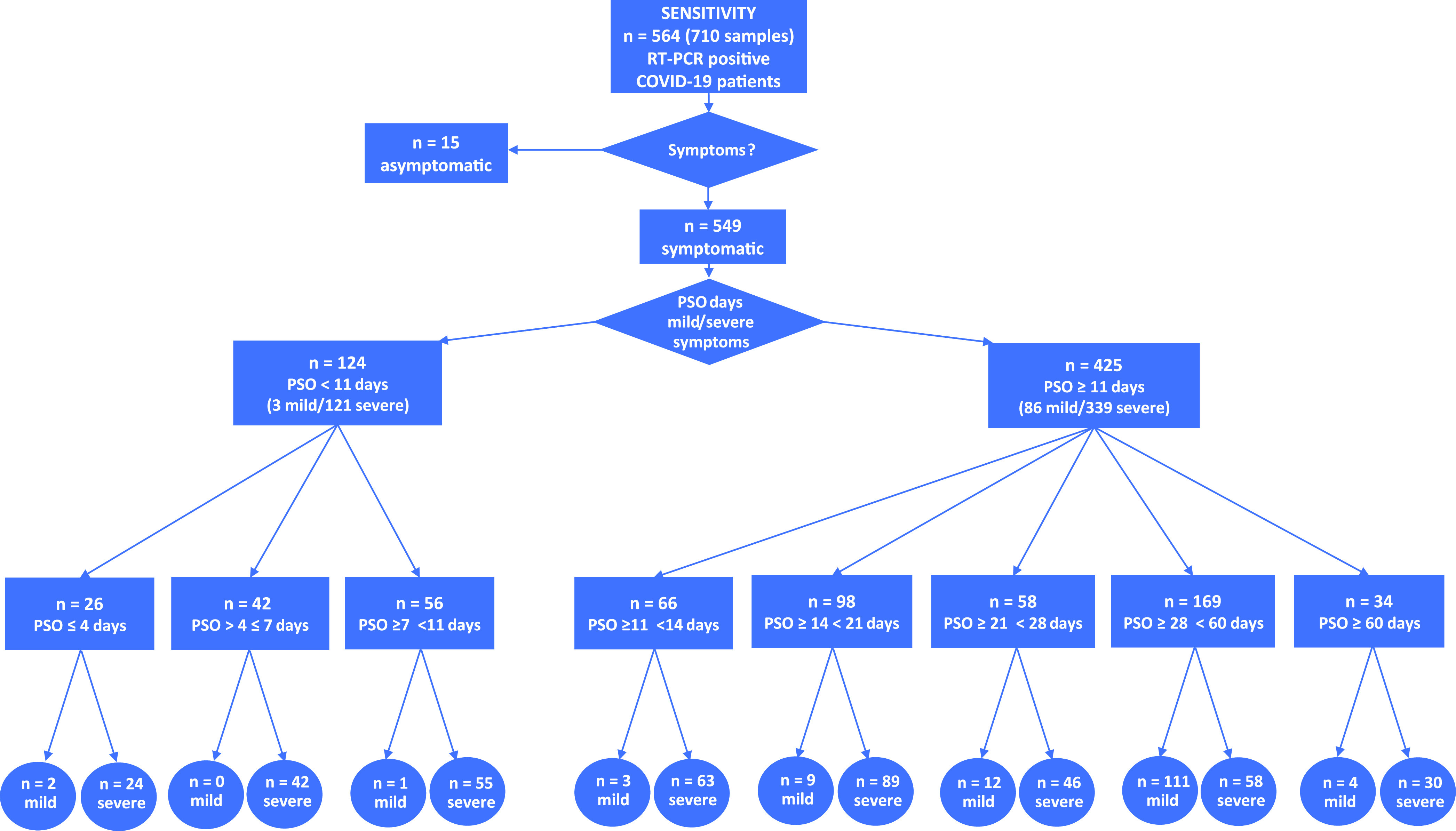
Flowchart of the serological study to test the clinical sensitivity of the BioSpeedia COVID19SEROSpeed IgM/IgG test. A total of 710 samples were used. Groups of SARS-CoV-2 RNA-positive enrolled patients (*n* = 564) were divided into asymptomatic and symptomatic cases and then subdivided based on disease severity and post-symptom onset (PSO) time of sampling. For a subgroup of symptomatic patients (*n* = 215), sensitivity was assessed on multiple sequential samples collected at different PSO times.

A total of 564 patients that had proven positive for SARS-CoV-2 RNA by qRT-PCR, composed of 549 symptomatic and 15 asymptomatic samples, were included for sensitivity analysis. Groups of symptomatic patients were classified according to the time (days) post-symptom onset (PSO) and divided into those with severe symptoms (those having required hospitalization) and those with mild symptoms (those kept at home) (Fig. 1). The serum specimens were collected from 17 February to 21 June 2020, in the course of hospitalization or after the end of the disease for mild cases; they were transported at room temperature within a few hours, centrifuged upon arrival to the laboratory, and stored at −30°C until use according to the standard procedure of each center. A total of 215 serum specimens sampled at different times from the same patient were available for 104 cases (*n* = 84 from Saint-Etienne, *n* = 19 from Zadar, and *n* = 1 from Brescia).

### Commercial tests used for performance comparison.

Six commercial tests were used to assess the performance of the BioS assay. A brief description of these tests together with the centers where they were used is given in Table 1. The tests were performed according to the recommendations of each manufacturer. The prepandemic specimens were not assayed by these tests.

**TABLE 1:** Characteristics of the serological tests that were used in this study^*a*^

Manufacturer	Test commercial name (abbreviation)	Viral target(s)	Antibody class(es)	Regulatory status	Technology	Site using the reagent
Abbott Laboratories	Abbott SARS-CoV-2 IgG (Abbott)	Nucleocapsid	IgG	CE-IVD, FDA	High-throughput CLIA	Saint-Etienne

Beijing Wantai Biological Pharmacy	Wantai SARS-CoV-2 Ab test (Wantai)	RBD	Total antibodies	CE-IVD	ELISA	Milan

BioSpeedia	COVID19SEROSpeed IgM/IgG (BioS)	S1 spike, nucleocapsid	IgM and IgG on separate lines	CE-IVD	Lateral flow assay	All sites

Diasorin	Liason SARS-CoV-2 S1/S2 (Diasorin)	S1 and S2 spike	IgG	CE-IVD, FDA	High-throughput CLIA	Saint-Etienne

Diesse Diagnostica	Enzywell SARS-CoV-2 IgG (Diesse IgG)	Native antigens from infected Vero E6 cells	IgG	CE-IVD	ELISA	Padua
	Enzywell SARS-CoV-2 IgM (Diesse IgM)	Native antigens from infected Vero E6 cells	IgM	CE-IVD	ELISA	Padua

Euroimmun	Anti-SARS-CoV-2 ELISA (IgG) (Euroimmun)	S1 spike (including RBD)	IgG	CE-IVD, FDA	ELISA	Bonn

a Abbreviations: CE-IVD, approved by the European Community for *in vitro* diagnostics; FDA, approved by the U.S. Food and Drug Administration; RBD, receptor binding domain of the spike protein; CLIA, chemiluminescence immunoassay; ELISA, enzyme-linked immunosorbent assay.

### Statistical analyses.

For the descriptive and analytical statistics, categorical variables were quantified as frequency rates and percentages. Continuous variables were quantified using means ± standard deviations (SD). Means for continuous variables were compared using independent group *t*-tests. Proportions for categorical variables were compared using the chi-square test or the Fisher exact test when the data were limited. Uncertainty in estimates of sensitivity and specificity was presented using 95% confidence interval (CI) data. Concordance with other commercial tests was calculated using overall concordance and Cohen’s kappa concordance test. All analyses were performed using Stata (Stata Corp., College Station, TX, USA), and *P* values of less than 0.05 were considered statistically significant.

## RESULTS

### Evaluation of the specificity of the BioS test.

The specificity of the BioS IgM-IgG test, assessed on 215 prepandemic serum specimens, was found to be 98.1% (95% CI, 96.2% to 99.4%). The four false-positive samples tested positive for IgG for 3 of them and IgM for 1 of them; 2 of these 4 patients (a 14-year-old boy and a 38-year-old woman) exhibited IgM directed toward Mycoplasma pneumoniae and Toxoplasma gondii, respectively.

### Overall characteristics of the subjects included in the evaluation of the clinical sensitivity of BioS.

A total of 710 serum specimens from SARS-CoV-2 RNA-positive patients available for this study were collected from 564 subjects (Fig. 1). As expected, the hospitalized patients (*n* = 460) were on average significantly older than the 89 subjects with mild symptoms and the 15 subjects who were asymptomatic (62.6 ± 16.1 versus 43.9 ± 17.7 years; *P < *0.001). Women were underrepresented among the hospitalized patients (33.5%; 95% CI, 0.26 to 0.41). Table S1 in the supplemental material reports patients’ demographic and clinical data per participating site. Tested on the 710 serum specimens represented in Fig. 1, BioS exhibited an overall clinical sensitivity of 86.0% (95% CI, 0.83 to 0.89) if at least one band (IgM or IgG) gave a positive result.

### Clinical sensitivity of specimens sampled prior to 11 days post-symptom onset.

As shown in Fig. 1, 124 patients were sampled prior to 11 days PSO (3 patients with mild symptoms and 121 hospitalized patients; 97 tested positive). The BioS test (IgM-positive and/or IgG-positive band) exhibited cumulative clinical sensitivities of 69.2% (18/26; 95% CI, 0.51 to 0.87) at ≤4 days PSO, 69.1% (47/68; 95% CI, 0.58 to 0.80) at ≤7 days PSO, and 78.2% (97/124; 95% CI, 0.71 to 0.86) at <11 days PSO. Of the 97 specimens sampled prior to 11 days PSO and found positive by the BioS test, 19 (19.6%) tested IgG positive but IgM negative, whereas 27 (27.8%) exhibited the opposite pattern (IgM only) and 51 (52.6%) were positive for both bands.

In 69 of the 124 patients tested early in the course of infection, all belonging to a center where the Wantai assay was being used (Table S1), the positive percent agreement of BioS was compared to that of this test, which measures total antibody response (IgM with or without IgG). The sensitivities of the two tests were similar (82.6% [95% CI, 0.80 to 0.89] for Wantai versus 78.2% [95% CI, 0.71 to 0.86] for BioS).

### Clinical sensitivity of specimens sampled ≥11 days post-symptom onset.

Of the sera collected from patients ≥11 days PSO (Fig. 1), 392/425 (92.2%), including 317/339 hospitalized patients (93.5%) and 75/86 patients with mild symptoms (87.2%), were positive for either IgM or IgG antibodies.

**FIG 2:**
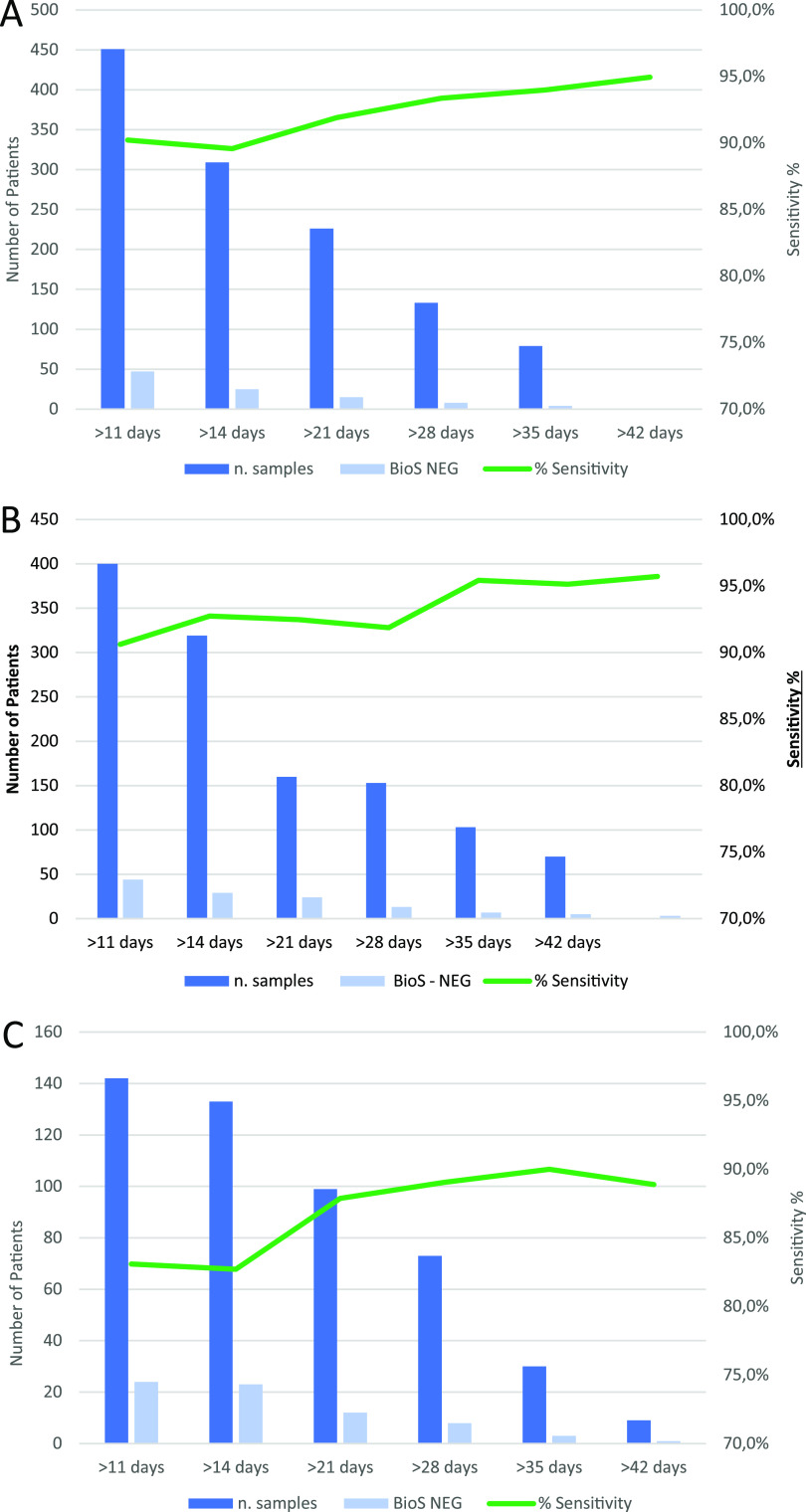
Clinical sensitivity of the BioSpeedia COVID19SEROSpeed IgM/IgG test (BioS), according to the time of sampling post-symptom onset. (A) All symptomatic patients. (B) Patients with severe disease (hospitalized). (C) Patients with mild disease. The dark blue bars correspond to the number of samples, the light blue bars to those found negative by the BioS test, and the green lines to the sensitivity of the BioS test at each time.

**FIG 3:**
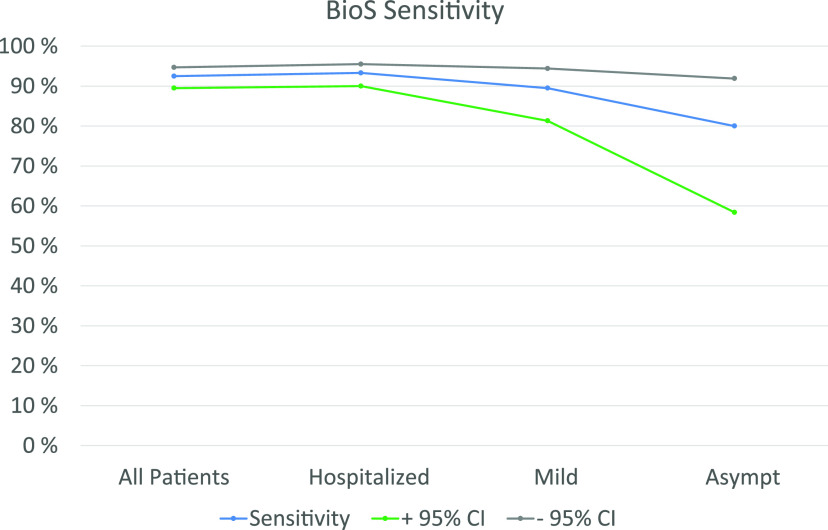
Clinical sensitivity with 95% confidence intervals of the BioSpeedia COVID19SEROSpeed IgM/IgG test according to the clinical status of patients on serum specimens taken at 11 days PSO or later.

Figure 2 illustrates the clinical sensitivity of BioS according to the time of sampling PSO and disease severity. Interestingly, the antibody prevalence increased much more rapidly in hospitalized patients than in those exhibiting a mild disease (Fig. 2B and C). The lowest sensitivity was seen in asymptomatic patients (86.4%; 95% CI, 0.69 to 1.00) (Fig. 3).

BioS exhibited positive percent agreement that was similar to or higher that seen with all the commercial tests used for comparison (Table 2).

**TABLE 2:** Positive percent agreement of antibody assays in RT-PCR-positive patients for SARS-CoV-2 sampled 11 days or more post-symptom onset^*a*^

Samples	COVID19SEROSpeed IgM/IgG test			Comparative assay		
	No. of positive results/total no. of results	PPA (%)	95% CI	No. of positive results/total no. of results	PPA (%)	95% CI
No comparative assay						
All patients	392/425	92.2	89.5–94.7			
Hospitalized	317/339	93.5	90.0–95.5			
Mild	75/86	87.2	81.3–94.4			

Abbott (Saint-Etienne)						
All patients	101/110	91.8	85.2–95.6	95/110	86.4	78.7–91.6
Hospitalized	66/70	94.3	86.2–97.8	64/70	91.4	82.5–96.0
Mild	35/40	87.5	73.9–94.5	31/40	77.5	62.5–87.7

Diasorin (Saint-Etienne)						
All patients	95/104	91.3	84.4–95.4	84/104	80.8	72.2–87.2
Hospitalized	63/67	94.0	85.6–97.7	57/67	85.1	74.7–91.7
Mild	32/37	86.5	72.0–94.1	27/37	73.0	57.0–84.6

Diesse IgG—Diesse IgM (Padua)						
All patients	57/62	91.9	82.5–96.5	56/62	90.3	80.5–95.5
Hospitalized	56/61	91.8	82.2–96.4	55/61	90.2	80.2–95.4
Mild	1/1	100	20.7–100	1/1	100	20.7–100

Euroimmun (Bonn)						
All patients	54/58	93.1	83.6–97.3	50/58	86.2	75.1–92.8
Hospitalized	19/19	100	83.2–100	18/19	94.7	75.4–99.1
Mild	35/39	89.7	76.4–95.9	32/39	82.1	67.3–91.0

Wantai (Milan)						
Hospitalized	62/65	95.4	86.9–98.4	46/65	70.8	57.6–79.8

a 95% CI, 95% confidence interval; PPA, positive percent agreement.

The overall concordance between BioS and the 5 comparators was evaluated by using the kappa-Cohen test (kCt). Good concordance was obtained with the Euroimmun assay (kCt of 0.62) and low concordance with the Wantai assay (kCt of 0.10); the three other tests had moderate concordance levels (kCt of 0.57, 0.47, and 0.41 for Abbott, Diasorin, and Diesse tests, respectively).

Of 34 patients sampled ≥60 days PSO (Fig. 1), 31 exhibited an antibody response with the BioS test, including 27 hospitalized and 4 mildly symptomatic patients. Most of them (*n* = 19) had IgG antibodies. The antibody band was faint in 11 cases. In 3 cases, no antibody response was seen.

### Kinetics of antibodies in individual patients.

Seven individuals for whom 4 successive serum specimens were available were tested to study the kinetics of the antibody response (Table 3). All individuals had severe disease requiring hospitalization. The BioS test was positive at the earliest date, either alone or along with some of the other assays, for 4 patients (A, C, D, and E), with IgM only for patients A and E. The Abbott test was positive earliest for patient B, and the Liaison test was positive earliest for patient F; BioS was positive for both of these patients at the next time interval. Patient G illustrates the absence of seroconversion by any test in relation to a severe immunosuppression due to a thymoma.

**TABLE 3:** Detection of SARS-CoV-2 RNA in nasopharyngeal samples by qRT-PCR and kinetics of the antibody response in 7 patients tested on 4 successive samples by 3 different assays^*a*^

ID (patient/sex/ yr of birth)	qRT-PCR (DPSO/ *C_T_* value)	Serum specimen result				
		DPSO	Bios		Diasorin (AU)	Abbott (AU)
			IgM	IgG		
A/male/1953	8/23.9	8	Negative	Negative	0.08	<3.8
		10	**Positive**	Negative	**1.4**	4.92
		17	**Positive**	**Positive**	**8.18**	**140**
		34	**Positive**	**Positive**	**9.03**	**300**
B/female/1949	5/14.9	11	Negative	Negative	0.3	**23.3**
		21	**Positive**	Negative	**1.85**	**102**
		37	**Positive**	**Positive**	**2.36**	**52.6**
		63	**Positive**	**Positive**	**5.56**	**87.9**
C/female/1935	2/20.8	3	Negative	Negative	0.03	7.88
		10	**Low positive**	**Low positive**	**6.23**	**35**
		12	**Low positive**	**Low positive**	**6.54**	**27.5**
		29	**Positive**	**Positive**	**9.36**	**88.6**
D/male/1954	5/17.5	7	Negative	Negative	0.01	<3.8
		10	Negative	Negative	0.18	6.76
		13	**Low positive**	**Positive**	**4.09**	**67.8**
		38	**Positive**	**Positive**	**9.7**	**183**
E/female/1933	0/33.1	0	Negative	Negative	0.09	<3.8
		9	**Low positive**	Negative	0.16	<3.8
		13	**Positive**	**Positive**	**3.66**	6.37
		19	**Positive**	**Positive**	**5.9**	14.2
F/male/1944	10/29.2	6	Negative	Negative	**2.89**	<3.8
		9	**Positive**	**Positive**	**8.92**	10
		12	**Positive**	**Positive**	**8.9**	**161**
		37	**Positive**	**Positive**	**9.63**	**182**
G/male/1981^*b*^	5/28.9	18	Negative	Negative	0.01	<3.8
		20	Negative	Negative	0.01	<3.8
		26	Negative	Negative	0.01	<3.8
		32	Negative	Negative	0.01	11

a The three assays used for testing were COVID19SEROSpeed IgM/IgG (Bios), Diasorin Liason SARS-CoV-2 S1/S2 (Diasorin), and Abbott SARS-CoV-2 IgG (Abbott). The thresholds of positivity were 1.4 and 15.0 for the Diasorin and Abbott tests, respectively. Bold characters correspond to positive results. “Low positive” corresponds to the presence of a faint band. ID, identifier; DPSO, days post-symptom onset; *C_T_* value, cycle threshold value for the PCR test; AU, arbitrary units.

b Subject G was a deeply immunocompromised patient presenting a thymoma.

## DISCUSSION

In this study, the BioS rapid test showed clinical specificity of 98.1% on a large panel of prepandemic serum specimens and clinical sensitivity of 92.5% in RT-PCR-confirmed COVID-19 patients sampled 11 days or more PSO. Moreover, in comparison with five commercial antibody assays, the BioS test exhibited excellent positive percent agreement in both severe and mild COVID-19 patients.

Evaluations of new serological tests based on LFA technology and comparisons to more-established methods targeting different classes of antibodies (IgM, IgA, and IgG) were addressed in several previous studies (15, 16, 18–22). The numerous biases affecting these studies were discussed and criticized in a recent meta-analysis (17). Our study was able to respond to some of these criticisms. In particular, (i) with regard to the number of cases, our study involved a large panel of specimens collected in different European hospitals and probably represents the largest currently reported in this kind of study; (ii) the sensitivity was evaluated on serum specimens taken exclusively from qRT-PCR-confirmed COVID-19 patients; (iii) the time from the onset of symptoms was known in all symptomatic cases; (iv) positive percent agreement data were compared by testing the same specimens with commercial assays using different viral targets; (v) a comparison could be done between patients with severe disease symptoms and those with mild symptoms and also asymptomatic subjects; (vi) for some patients, sequential serum specimens were available to study the kinetics of the antibody response.

In more than 78% of tested specimens, the rapid test allowed the demonstration of antibodies to SARS-CoV-2, even in sera collected before the day 11 from the onset of symptoms, i.e., before the time expected for seroconversion. Interestingly, among samples collected before day 11, 19.6% of them had only an IgG response. As in most viral diseases, IgM was reported to appear before IgG in an early study (12), but more-recent studies reported IgG seroconversion that occurred simultaneously with or even earlier than IgM seroconversion, which remained undetectable in many cases (23, 24), as observed in the past with SARS patients (25). Our results support those findings.

Unlike most serological tests currently available, BioS allows separate detection of IgM and IgG antibodies, which may improve the sensitivity of COVID-19 diagnosis in recent infections with regard to the use of molecular tests only (12, 13). A further explanation of the good sensitivity shown before day 11 by the rapid test is that it addresses antibodies directed against both S1 and N viral proteins, while many comparators are directed against a single target. The best concordance level (0.91)—with a Cohen’s kappa coefficient of 0.62—was observed with the Euroimmun assay, which targets the S1 protein, including the receptor binding domain (RBD) of the spike protein that was previously shown to correlate to neutralizing antibodies (26). In samples collected <11 days PSO, the positive percent agreement of the BioS assay (78.2%) was very close to that of the Wantai assay (82.6%).

Previous studies suggested that patients with the mild form of COVID-19 tend to have a delayed antibody response compared to severely ill hospitalized patients (12, 26, 27). Our results confirm these observations. The absence of antibody response or the presence of a faint signal in a few subjects at the late phase of infection is consistent with previous reports and has been interpreted as a possible consequence of virus-induced immunosuppression. Its greater frequency in patients with mild infection would, however, suggest poor immunogenicity of the virus in this context. Among the subjects included in our study who developed no response during the whole course of the disease, a 70-year-old patient who had been hospitalized for severe COVID-19 was affected by thymoma, which probably explains the absence of specific antibodies (see patient G in Table 3).

Finally, on the basis of data from 32 patients who were sampled ≥60 days PSO, we observed that IgG levels likely start to decrease within 2 to 3 months after infection. Long et al. (27) reported similar findings and referred to another analysis of the dynamics of neutralizing antibody titers in asymptomatic patients with COVID-19, where 50% of patients showed decreased neutralizing antibodies approximately 6 to 7 weeks PSO. The same pattern was reported recently in symptomatic patients (28).

Our study had some limitations. First, only the BioS assay was tested on all samples, while the other assays were used with different subsets of samples according to their availability. Second, although PSO is a key factor in the analysis of sensitivity results, the accuracy of this patient-reported information is likely limited, notably in mild infections. Third, with the exception of the Diesse and Wantai tests, which detect IgM and IgG antibodies on the same band, none of the assays used in this study detected antibodies belonging to classes other than IgG, which could explain the moderate or weak concordance of the BioS test with most of the other tests. Finally, our study targeted mainly hospitalized patients with severe disease; the number of subjects with mild symptoms and asymptomatic subjects was small, which impairs the scope of our evaluation.

In conclusion, the BioS rapid test was shown to be specific and sensitive for the accurate detection of anti-SARS-CoV-2 antibodies in different clinical settings within 15 min, with a good sensitivity even in the very early phases of the infection. Furthermore, the presence of an IgM-independent band, documenting a possible recent infection, in association with molecular testing, may contribute to the diagnosis of COVID-19. Our results also confirm recent findings underlining the possibility of an earlier IgG anti-SARS-CoV-2 response and the need to also test patients positive for IgG alone by qRT-PCR assay to verify their infectiousness. Our results also demonstrate the need of appropriate timing of sample collection in acutely ill COVID-19 patients.

## Supplementary Material

Supplemental file 1
