# Supplementary material for: Performance of the COVID19SEROSpeed IgM/IgG Rapid Test, an Immunochromatographic Assay for the Diagnosis of SARS-CoV-2 Infection: a Multicenter European Study
Source: J Clin Microbiol. 2021 Jan 21;59(2):e02240-20. doi: 10.1128/JCM.02240-20 (PMC8111158; doi:10.1128/JCM.02240-20)
Supplement: Supplemental file 1 [file JCM.02240-20-s0001.pdf]

**Supplementary Materials**

**Table S1**. Demographics and disease status of patients included in the analysis, per site.

| Site (country) | N | Age | | Females | | Disease status | | | | | | | |
|---|---|---|---|---|---|---|---|---|---|---|---|---|---|
| | | | | | | Asymptomatic | | Mild | | Hospitalized | | | |
| | | Mean | SD | N | % | N | % | N | % | N | % | Age mean | SD |
| Brescia (Italy) | 75 | 64.3 | 13.2 | 35 | 46.7 | 0 | 0 | 0 | 0 | 75 | 100 | 64.3 | 13.2 |
| Milano (Italy) | 175 | 56.2 | 15.9 | 57 | 32.6 | 0 | 0 | 0 | 0 | 175 | 100 | 56.2 | 15.9 |
| Padua (Italy) | 115 | 59.6 | 19.8 | 36 | 31.3 | 6 | 23.8 | 6 | 4.2 | 103 | 89.6 | 65.7 | 15.4 |
| Saint-Etienne (France) | 139 | 60.9 | 20.7 | 63 | 45.3 | 9 | 6,5 | 43 | 30.9 | 87 | 62.6 | 71.2 | 13.9 |
| Zadar (Croatia) | 60 | 51.9 | 17.4 | 37 | 61.7 | 0 | 0 | 40 | 66.7 | 20 | 33.3 | 58.4 | 18.5 |
| **Total** | **564** | **58.7** | **17.7** | **228** | **40.4** | **15** | **7.3** | **89** | **15.2** | **460** | **81.6** | **62.5** | **16.9** |
